# *Cheiloneurus flaccus* (Walker, 1847) (Hymenoptera: Encyrtidae), new to New Zealand

**DOI:** 10.3897/BDJ.1.e958

**Published:** 2013-09-16

**Authors:** Stephen E. Thorpe

**Affiliations:** † School of Biological Sciences (Tamaki Campus), University of Auckland, Auckland, New Zealand

**Keywords:** *Cheiloneurus flaccus*, Hymenoptera, Encyrtidae, New Zealand, Auckland, Dryinidae, NZOR

## Abstract

*Cheiloneurus flaccus* (Walker, 1847) is reported from New Zealand for the first time.

## Introduction

*Cheiloneurus flaccus* a distinctive species of encyrtid found widely in the New World, and also Australia (Guerrieri 2006). It is a parasitoid of dryinids. It is herein newly recorded from Auckland, New Zealand.

## Taxon treatments

### Cheiloneurus flaccus

(Walker, 1847)

#### Materials

- **Type status:** Other material. **Occurrence:** recordedBy: Stephen Thorpe; individualCount: 2; sex: female; **Location:** country: New Zealand; verbatimLocality: Tamaki Campus of University of Auckland; verbatimLatitude: 36.88661S; verbatimLongitude: 174.85253E; **Event:** eventDate: 20 April 2013; **Record Level:** institutionCode: Auckland Museum

#### Description

**Figure 1.**
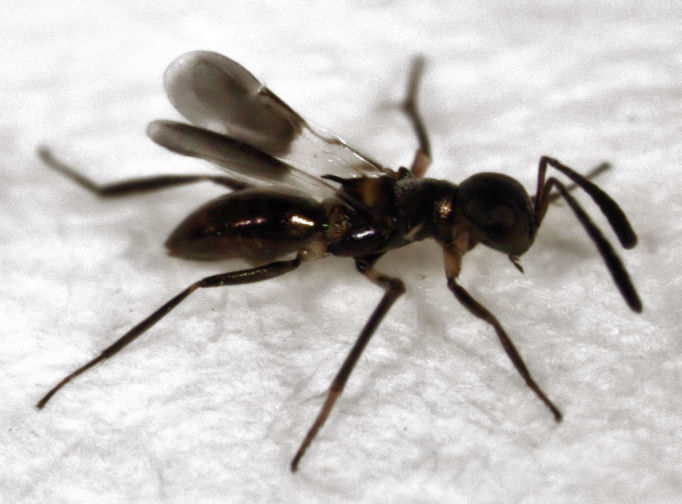
*Cheiloneurus flaccus* (female), length about 2 mm

**Figure 2.**
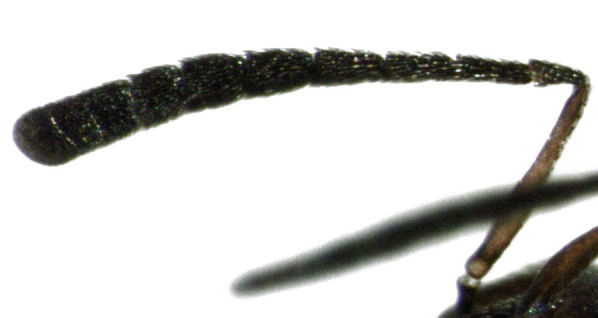
*Cheiloneurus flaccus*, female antenna

On 20 April 2013, while looking for insects in swards of long grass on the Tamaki Campus of the University of Auckland, I noticed two specimens of an unusual looking encyrtid. I collected one of the two specimens (see Figs 1, 2), and identified it as *Cheiloneurus flaccus*, using the redescription by Guerrieri and Viggiani (2005). *Cheiloneurus flaccus* is a distinctive species, quite different to the two congeneric species already known from N.Z. The antenna, in particular, is distinctive, with the first segment of the funicle much longer than the pedicel (see Fig. 2). The species is known to be a parasitoid of dryinids. The site where I found *Cheiloneurus flaccus* also abounds with dryinids belonging to two species of *Gonatopus*. Two other species of dryinid (*Bocchus thorpei*, and *Dryinius koebelei*) also occur on the campus. I recommend that *Cheiloneurus flaccus* be added to the New Zealand Organisms Register (NZOR) as exotic, present in the wild.

## Supplementary Material

XML Treatment for Cheiloneurus flaccus
